# Multi-Objective Optimization of Acoustic Performances of Polyurethane Foam Composites

**DOI:** 10.3390/polym10070788

**Published:** 2018-07-18

**Authors:** Shuming Chen, Wenbo Zhu, Yabing Cheng

**Affiliations:** 1 State Key Laboratory of Automotive Simulation and Control, Jilin University, Changchun 130022, China; smchen@jlu.edu.cn (S.C.); zwbzjjjack@gmail.com (W.Z.); 2 School of Mechanical and Aerospace Engineering, Jilin University, Changchun 130022, China

**Keywords:** grey relational analysis, multi-objective particle swarm optimization, acoustic performances, Ethylene Propylene Diene Monomer, polyurethane foam composites

## Abstract

Polyurethane (PU) foams are widely used as acoustic package materials to eliminate vehicle interior noise. Therefore, it is important to improve the acoustic performances of PU foams. In this paper, the grey relational analysis (GRA) method and multi-objective particle swarm optimization (MOPSO) algorithm are applied to improve the acoustic performances of PU foam composites. The average sound absorption coefficient and average transmission loss are set as optimization objectives. The hardness and content of Ethylene Propylene Diene Monomer (EPDM) and the content of deionized water and modified isocyanate (MDI) are selected as design variables. The optimization process of GRA method is based on the orthogonal arrays $L_{9}\left(3^{4}\right)$, and the MOPSO algorithm is based on the Response Surface (RS) surrogate model. The results show that the acoustic performances of PU foam composites can be improved by optimizing the synthetic formula. Meanwhile, the results that were obtained by GRA method show the degree of influence of the four design variables on the optimization objectives, and the results obtained by MOPSO algorithm show the specific effects of the four design variables on the optimization objectives. Moreover, according to the confirmation experiment, the optimal synthetic formula is obtained by MOPSO algorithm when the weight coefficient of the two objectives set as 0.5.

## 1. Introduction

Our living and working environment has been gradually perplexed by noise pollution due to the rapid developments of modern industries and transportations. Vehicle noise is a major source of noise pollution, which consists of interior noise and exterior noise. Recently, vehicle interior noise is becoming one of the important indices for quality evaluation of vehicles because it not only imposes danger on drivers and passengers’ health, but also decreases the comfort of driving [1,2]. Therefore, with the development of social transportation, eliminating vehicle interior noise has involved current and broad interests of automobile manufacturers. The use of acoustic package is an effective method to reduce vehicle interior noise. Thus, the acoustic package design of automotive has become an important research for the automobile industry.

There are mainly two kinds of acoustic package materials: sound absorption materials and sound insulation materials. Sound insulation materials have a high surface density and can reflect sound energy to the incident direction. However, the sound absorption materials are light and have a high porosity, which makes the acoustic wave easily accessible to the interior of the materials [3]. It means that the sound absorption ability and sound insulation ability of acoustic package materials are hard to get the maximum value simultaneously. Polyurethane (PU) foam is a kind of effective sound absorption material in automobile industry due to the effective sound damping and low-density characteristics. It has been widely applied in interior components, such as seats, inner dash mats, and other acoustic trim parts. The acoustic wave propagation in PU foams mainly dissipates as viscous friction on interconnected pores and thermal heat exchange on solid-fluid boundary [4]. However, pure PU foam only shows great sound absorption ability in high frequency region due to the special pore morphologies. Previous studies have shown that the acoustic performances of PU foams can be modified by adding functional particles to PU foams or adjusting the chemical compositions of PU foams [5,6,7,8,9,10,11]. However, it not only cannot get the optimum acoustic performances, but also cause the waste of materials, if the materials are simply mixed together. Thus, this paper improves the acoustic performances of PU foam composites by optimizing the synthetic formula.

Recently, many researchers put their efforts to improve the acoustic performances of acoustic package materials by different optimization methods. Jeon et al. [12] used particle swarm optimization (PSO) algorithm for optimal bending design of vibrating plate to minimize noise radiation. Chen et al. [13] applied the grey rational analysis (GRA) with Taguchi method to optimize the acoustic performances of the sound package. Jiang et al. [14] employed the Taguchi method base on orthogonal arrays to conduct the experiments to improve the acoustic behaviors of PU foams. He et al. [15] utilized the GRA method and multi-objective particle swarm optimization (MOPSO) algorithm to optimize the acoustic package materials of firewall and floor. Pan et al. [16] dealt with the optimization of the sound package by using a genetic algorithm to satisfy acoustical targets and packaging requirements in the vehicle design process. Grubeša et al. [17] applied the genetic algorithm to optimize the acoustic performances and economic feasibility of barrier cross section. The materials and cross section shapes of the barrier are considered in the optimization process. Kim et al. [18] applied acoustic topology optimization for sound barrier with rigid and porous materials by the finite element method. Considering the sound absorption ability and sound insulation ability are equally important to reduce vehicle interior noise. Both of them should be simultaneously maximized, which is a multi-objective optimization problem inherently. Therefore, this paper applies multi-objective optimization method to optimize the synthetic formula of PU foam composites.

The MOPSO algorithm is one of the evolutionary algorithms that based on the social behavior of flocks of birds that adjust their movement to find the best food position. It has been widely and prevalently applied to solve engineering problems in different fields due to the advantages of relatively fast convergence and good handle continuous, discrete, and integer variables types [19,20,21]. Normally, the analysis models of the acoustic performances of acoustic package materials are complicated, and the normal optimization processes are of extremely low optimization efficiency. In contrast, the surrogate models are more efficient and they can easily bridge the gap among multi-objective optimization. Therefore, it has been widely applied in multi-objective optimization design [21]. On the other hand, GRA method is a branch of grey system theory, which can be effectively used to analyze the complicated interrelationship among the designated performance characteristics. By combing the entire range of performance criterion values into a quantified value of grey relational grade (GRG). It can be used to identify the major influencing factor and the dominant or subordinate relationship from various factors of multi criteria problems [22,23,24,25,26]. Therefore, both the GRA method and MOPSO algorithm are applied in this paper to optimize the formulation of PU foam composites for good acoustic performances. The optimization process of MOPSO algorithm is based on the surrogate model, and the optimization process of GRA method is based on the orthogonal arrays.

A previous study shows that the acoustic performances of PU foam composites are changed when filled with Ethylene Propylene Diene Monomer (EPDM) of different content and hardness. Meanwhile, in the synthesis process of PU foam, modified isocyanate (MDI) is a matrix material and deionized water is used as blowing agent. Both have an impact on the acoustic performances by changing the pore morphologies and the density of PU foams. Moreover, the acoustic performances of PU foam composites can be evaluated by the sound absorption coefficient and sound transmission loss. Therefore, the sound absorption coefficient and sound transmission loss of PU foam composites are investigated in this paper by changing the content of MDI and deionized water, the content and hardness of EPDM. The aim of this paper is to obtain a synthetic formula of a PU foam composite with high sound absorption ability and sound insulation ability under the condition that the sound absorption ability and sound insulation ability are equally important for reducing vehicle interior noise. Therefore, this paper uses the GRA method and MOPSO algorithm to optimize the synthetic formula of PU foam composites, and then the actual samples are prepared according to the optimization results for comparison to determine the optimal synthetic formula. Meanwhile, synthetic formula optimization can improve the utilization rate of PU foam composites preparation materials and reduce environmental pollution that is caused by waste EPDM.

## 2. Materials and Methods

### 2.1. Materials

In this paper, PU foam is synthesized using MDI and polyether polyols by a one-step polymerization process. The polyether polyols include 330 N (OH-value: 33–36 mg KOH/g) and 3630 (OH-value: 33–37 mg KOH/g). MDI (diphenylmethane 4, 4-diisocyanate,) is used as matrix material. A1 (mixture of 70% 2-dimethylaminoethyl ether and 30% dipropylene glycol), A33 (solution of 33% triethylenediamine) and Tri-ethanolamine (TEA) are chosen as the catalysts for the gelling reaction. Silicone oil is used as the surfactant. Deionized water is used as a blowing agent to produce CO_2_ gases and amine functionalities. The TEA is obtained from Guangdong Wengjiang Chemical Reagent Co., Ltd., Guangdong, China. The other chemical materials are obtained from Jining Huakai Resin Co., Ltd., Shandong, China. EPDM is used as a functional particle introduced into PU foams. The EPDM of the same size have three different hardness: 65, 70 and 85 HA. It is obtained from Dongguan Zhangmutou Hongfa Plastic Raw Materials Business Department, Guangdong, China.

### 2.2. Sample Preparation

The materials except for MDI and EPDM are gradually weighed in a paper cup and pre-mixed at 1500 rpm for 60 s by using a mechanical mixer equipped with two impellers. Secondly, the various EPDM are added to the mixtures separately and stirred for 30 s. Finally, MDI is added to this mixture and stirred for 15 s. Then, the mixture is poured rapidly into mold. After curing 30 min at 50 °C in drying oven, the foams are removed and saved at room temperature for 24 h. Table 1 shows the raw materials used to prepare pure PU foams.

### 2.3. Experiment Design

In this paper, the content and hardness of EPDM, the content of MDI and deionized water are selected as design variables. The average sound absorption coefficient and average transmission loss are selected as the optimization objectives. The paper aims to simultaneously maximize the average sound absorption coefficient and average sound transmission loss. Table 2 lists the four design variables and their levels. The content level of MDI and deionized water are selected according to their function in the synthesis process. The content and hardness level of EPDM are selected according to the engineering experiences.

The first two columns of Table 3 show the details of the experiment schemes. In order to reduce the number of experiments and satisfy the requirements of the surrogate models, the 15 experimental samples are prepared in this paper. The first nine experimental samples are obtained by the orthogonal arrays $L_{9}\left(3^{4}\right)$, which are used for the optimization process of GRA method. The other samples are obtained by random selection. All of the experimental data are used to construct the surrogate models for MOPSO algorithm.

### 2.4. Measurement Method

The sound absorption coefficient of PU foam is defined as the ratio of the absorbed acoustic energy to the incident acoustic energy. However, the sound absorption coefficient is different with the frequency change. Therefore, the average sound absorption coefficient is widely used in order to evaluate the sound absorption ability in engineering practice. In this paper, the average sound absorption coefficient is the average value of the sound absorption coefficient at the 1/3 octave band on the 100–4000 Hz frequency band. It is calculated with Equation (1):

(1) $$\alpha_{a}=\frac{\alpha_{100}+\alpha_{125}+\cdots +\alpha_{3150}+\alpha_{4000}}{6}$$

where $\alpha_{a}$ represents the average sound absorption coefficient. $\alpha_{100}∼\alpha_{4000}$ represent the sound absorption coefficients at 100, 125, …, 3150, and 4000 Hz, respectively.

Sound transmission loss, as an inherent characteristic of acoustic package materials, can be used to evaluate the sound insulation ability. The average transmission loss is the average value of the transmission loss at the 1/3 octave band on the 100–4000 Hz frequency band. It is expressed as Equation (2):

(2) $$TL_{a}=\frac{TL_{100}+TL_{125}+\cdots +TL_{3150}+TL_{4000}}{17}$$

where $TL_{a}$ represents the average transmission loss. $TL_{100}∼TL_{4000}$ represent the transmission loss at frequencies of 100 Hz, 125 Hz, …, 3150 Hz, and 4000 Hz, respectively.

The sound transmission loss and sound absorption coefficient of PU foam composites are measured with SCS90AT acoustic materials properties measurement system (SCS, Padova, Italy), which is based on the standard of ISO 10534-2:2009(E) [27]. The sound absorption coefficient is obtained by using a two-microphone impedance tube, and the sound transmission loss is measured by four-microphone impedance tube. Cylindrical 30 mm thickness samples with 100 and 28 mm in diameters are tested for the frequency ranges of 100–1500 Hz and 500–6300 Hz, respectively. Then, the average sound absorption coefficient and average transmission loss are calculated through the equations. The results are shown in the latter two columns of Table 3.

## 3. Results

### 3.1. GRA Method

#### 3.1.1. Grey Relational Generation

Firstly, the first nine experimental results in the latter two columns of Table 3 are transformed to dimensionless sequences. The linear normalization preprocess is using the larger-the-better criterion with Equation (3):

(3) $$x_{i}^{*}\left(k\right)=\frac{x_{i}^{0}\left(k\right)-minx_{i}^{0}\left(k\right)}{maxx_{i}^{0}\left(k\right)-minx_{i}^{0}\left(k\right)}$$

where $x_{i}^{*}\left(k\right)$ denotes the normalized value of the ith value in the kth origin sequence. $x_{i}^{0}\left(k\right)$ denotes the original value of the ith value in the kth origin sequence. $\max x_{i}^{0}\left(k\right)$ and $\min x_{i}^{0}\left(k\right)$ represent the maximum and minimum values of the kth origin sequence, respectively. k is the number of quality characteristics. i is the row label of the experiments.

The average sound absorption coefficient and average transmission loss of the PU foam composites are set into origin sequence $x_{0}^{*}\left(k\right)=1$, *k* = 1, 2. The second column of Table 4 shows the results of the normalized sequences.

#### 3.1.2. Grey Relational Coefficient

After grey relational generation, the grey relational coefficient (GRC) is calculated with Equation (4). A high GRC reflects an intense relation between the origin sequence and the normalized sequence.

(4) $$\{\begin{array}{c} \varepsilon_{i}\left(x_{0}^{*}\left(k\right),x_{i}^{*}\left(k\right)\right)=\frac{\Delta_{min}+\zeta \cdot \Delta_{max}}{\Delta_{0i}\left(k\right)+\zeta \cdot \Delta_{max}}\\ \Delta_{0i}\left(k\right)=\|x_{0}^{*}\left(k\right)-x_{i}^{*}\left(k\right)\|\\ \Delta_{min}=\begin{array}{c} min\\ \forall i \end{array}\begin{array}{c} min\\ \forall k \end{array}\Delta_{0i}\left(k\right)\\ \Delta_{max}=\begin{array}{c} max\\ \forall i \end{array}\begin{array}{c} max\\ \forall k \end{array}\Delta_{0i}\left(k\right) \end{array}$$

where $\varepsilon_{i}\left(x_{0}^{*}\left(k\right),x_{i}^{*}\left(k\right)\right)$ denotes the GRC. $x_{0}^{*}\left(k\right)$ is the origin sequence. $x_{i}^{*}\left(k\right)$ is the normalized sequence. $\Delta_{0i}\left(k\right)$ is the deviation sequence of $x_{i}^{*}\left(k\right)$ and $x_{0}^{*}\left(k\right)$. ζ is the distinguishing coefficient.

In this paper, ζ is selected as 0.5 because the sound absorption ability and the sound insulation ability are equally important to reduce noise. Meanwhile, it brings higher identification degree between the two objectives [28]. The results are shown in the third column of Table 4.

#### 3.1.3. GRG

GRG is an average sum of GRC and calculated with Equation (5). The results are listed in the last column of Table 4.

(5) $$\gamma_{i}\left(x_{0}^{*},x_{i}^{*}\right)=\overset{n}{\underset{k=1}{\sum }}w_{k}\varepsilon_{i}\left(x_{0}^{*}\left(k\right),x_{i}^{*}\left(k\right)\right)$$

where $\gamma_{i}\left(x_{0}^{*},x_{i}^{*}\right)$ denotes the GRG. $\overset{n}{\underset{k=1}{\sum }}w_{k}=1$, $w_{k}$ is the weight of the *k*th quality characteristic. *n* is the number of performance characteristics. In this paper, $w_{k}$ is set as 0.5 and *n* is 2.

In the GRA method, GRG shows the relation between the origin and normalized sequences. Meanwhile, the average GRG can be used to evaluate the influence degree of design variables on objectives. It is calculated with Equation (6) and the results are shown in Table 5.

(6) $$GRG_{a}=\frac{\sum_{i=1}^{n}GRG_{i}}{n}$$

where $GRG_{a}$ is the average GRG of each level for different variables. i denotes ith level of the variables. $GRG_{i}$ is the GRG of ith level of the variables. n is the level numbers of the variables.

It can be seen in Table 5 that the biggest average GRG of the four design variables is 0.569, 0.633, 0.561, and 0.565, respectively. They are corresponding to level 3, level 1, level 1, and level 3 of the four design variables, respectively. It indicates that the PU foam composite has good acoustic performances when the content of MDI is 32 g, the content of EPDM is 2 g, the hardness of EPDM is 65 HA, and the content of deionized water is 3.5 g. On the other hand, the column of “Range” in Table 5 denotes the deviation between the maximum $GRG_{a}$ and the minimum $GRG_{a}$ of the same variable. The bigger range means the variable has a significant influence on acoustic performances of PU foam composites [29]. Therefore, the influence degree of the four design variables on the optimization objectives can be determined through comparing the range in Table 5. It can be observed that the biggest range is 0.13 for variable B and the smallest range is 0.034 for variable C. Thus, the order of influence of the design variables is B > A > D > C. Accordingly, the content of EPDM has a significant influence on the acoustic performances of PU foam composites and the hardness of EPDM has the least influence. It means that the small change of the content of EPDM will cause a large change in acoustic performances of PU foam composites. Meanwhile, variables A and D are the chemical compositions of PU foams and the range is very close. It indicates that the two variables have a similar influence level on the acoustic performances of PU foam composites.

### 3.2. MOPSO Algorithm

#### 3.2.1. Surrogate Model

In this paper, in order to select an appropriate surrogate model to express the relation between design variables and optimization objectives, the Response Surface (RS) and Kriging and Radial Basis Function Neural Network (RBFNN) methods are first separately employed to construct the surrogate models. Then, the better surrogate model is selected based on the fitting accuracy of the three models. To evaluate the fitting accuracy of the surrogate models, such coefficients as Determination Coefficient (DC), Relative Average Absolute Error (RAAE), and Relative Maximum Absolute Error (RMAE) are adopted [30]. The accuracy of the models is evaluated by another five random points in Table 6.

The evaluation coefficients of the surrogate models are listed in Table 7. It can be found that the DC of RS model of the average sound absorption coefficient and average transmission loss is 0.9507 and 0.9653, respectively. Both are the biggest and more than 0.95. Besides, the RAAE and RMAE values of RS model are the smallest. In general, the higher DC values, the more accurate the approximation models. The smaller the RAAE and RMAE values, the better the metamodel [30]. Thus, the RS model has better fitting accuracy than the Kriging model and the RBFNN model in this paper. Therefore, the RS model is adopted to construct the complex mapping between the optimization objectives and design variables.

#### 3.2.2. MOPSO Process

The formulation of the MOPSO algorithm in this paper is expressed as Equation (7):

(7) $$\{\begin{array}{c} Find\;:X=\left(X_{A},X_{B},X_{C},X_{D}\right)\\ Max\;:\;Y=\left\{y_{1}\left(X\right),\;y_{2}\left(X\right)\right\}\\ Subject\;to:\{\begin{array}{c} 28\leq X_{A}\leq 32\\ 2\leq X_{B}\leq 6\\ X_{C}=\left[65,70,85\right]\\ 2.5\leq X_{D}\leq 3.5 \end{array} \end{array}$$

where $X_{A},X_{B},X_{C}$, and $X_{D}$ are the design variables, which represent the content of MDI, the content of EPDM, the hardness of EPDM, and the content of deionized water, respectively. $y_{1}\left(X\right)$ and $y_{2}\left(X\right)$ represent the average sound absorption coefficient and average transmission loss, respectively.

**Figure 1:**
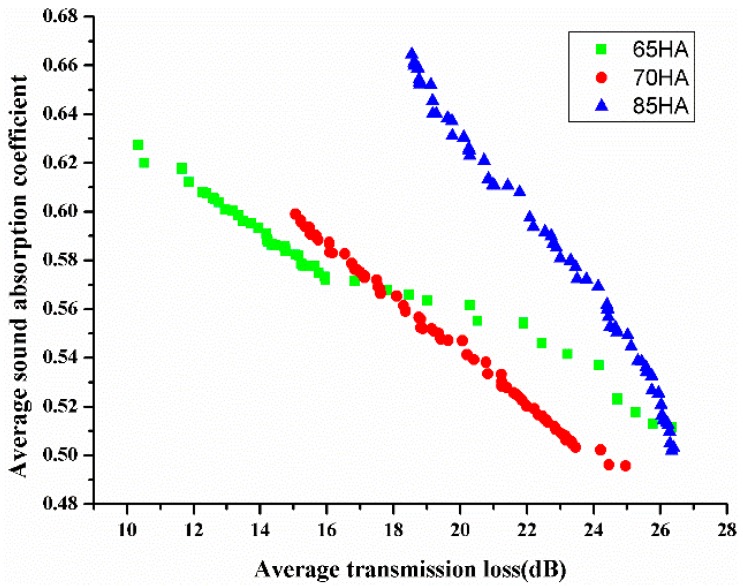
Pareto solutions of Response Surface (RS) model.

In MOPSO algorithm, the inertia weight coefficient is set as 0.5 to achieve a balance exploration and development capability. The particle increment is selected as 0.9, which represents the weight coefficient of the particle tracks its best solutions. The global increment is selected as 0.9, which represents the weight coefficient of the particle tracks the best solutions for the group. To obtain better convergence and faster calculation speed, the total number of particles and the maximum iteration are set as 10 and 300, respectively. Both the failed run penalty value and the objective value are set as 1 × 10^−30^. However, the hardness of EPDM in this paper is a discrete value, and only 65, 70, and 85 HA can be selected. Besides, the MOPSO algorithm is a kind of stochastic algorithm. It means the several runs have been performed before the good distribution uniformity of Pareto fronts is obtained. Finally, the Pareto optimal solutions of each EPDM are obtained, as shown in Figure 1. Both sound absorption ability and sound insulation ability of PU foam composites are impacted by the hardness of EPDM. Meanwhile, the average sound absorption coefficient of the PU foam composites has an opposite trend to the average transmission loss. The better the sound absorption ability of PU foam composites, the worse the sound insulation ability. It agrees with the actual situation. Note that the sound absorption ability and sound insulation ability are equally important in this paper. According to rank the Pareto optimal solutions from best to worst, the optimum values of the design variables are obtained. The best combination values of the variables are MDI of 32 g, deionized water of 3.4 g, EPDM of 5.8 g, and the hardness of EPDM is 65 HA.

#### 3.2.3. Analysis of MOPSO Results

**Figure 2:**
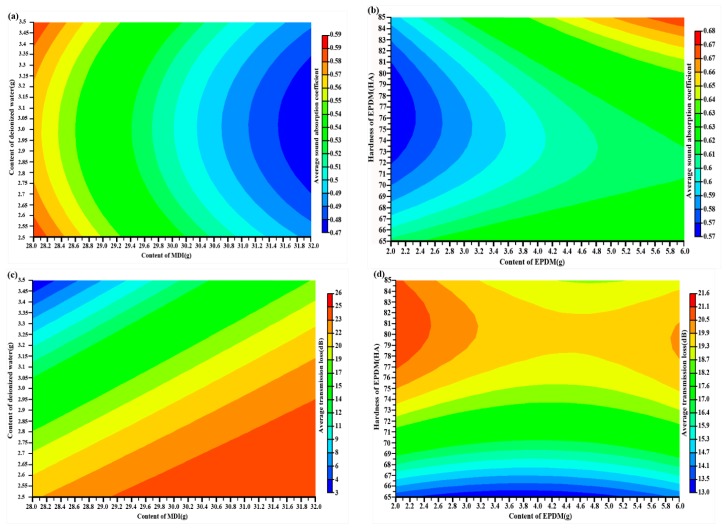
(**a**) Effect of chemical compositions on sound absorption ability; (**b**) Effect of functional particle on sound absorption ability; (**c**) Effect of chemical compositions on sound insulation ability; and (**d**) Effect of functional particle on sound insulation ability.

In this paper, EPDM is used as a functional particle to add to PU foam and not reacting with the chemical compositions. Therefore, the interaction between the design variables exists only in chemical compositions or functional particle. Figure 2 shows the specific effects of the four design variables on sound absorption ability and sound insulation ability.

The effects of the four design variables on sound absorption ability of PU foam composites are shown in Figure 2a,b. When considering one variable at a time, Figure 2a shows that the content of MDI has a larger influence on sound absorption ability than the content of deionized water on sound absorption ability. The average sound absorption coefficient of PU foam composites is higher when the content of MDI is taken the smaller value. The content of deionized water is located at the two ends of the range also be advantageous for improving the sound absorption ability. Figure 2b shows the average sound absorption coefficient of PU foam composites is higher when the content of EPDM is taken the bigger value within the range of this paper. However, when the hardness of EPDM is taken the intermediate value within the range of this paper, the average sound absorption coefficient of PU foam composites is low. Figure 2c,d show the effects of the four design variables on the sound insulation ability of PU foam composites. In Figure 2c, when the content of deionized water is smaller and the content of MDI is bigger, the average transmission loss of PU foam composites is higher. Meanwhile, it also can be observed that the content of deionized water has a significant influence on the sound insulation ability. When considering one variable at a time, it can be found in Figure 2d that the sound insulation ability is higher when the content of EPDM is located at two ends within the range of this paper. However, when the hardness of EPDM is taken the bigger value in the range of this paper, the sound insulation ability is better. On the other hand, it can be found in Figure 2 that the same values of chemical compositions and functional particle have opposite influence on sound absorption ability and sound insulation ability. Therefore, to get the optimum sound absorption and sound insulation ability simultaneously, the content and hardness of EPDM and the content of MDI and deionized water should be taken the compromise values within the range of this paper.

## 4. Verification

Once the optimal formulation of the PU foam composites is determined, it is important to verify whether the results of the optimization methods are appropriate. The optimum PU foams are not included in the prepared samples. Thus, the validation samples are prepared, according to the optimization results of the GRA method and MOPSO algorithm, respectively. Then, the transmission loss and sound absorption coefficient are measured. After that, the average transmission loss and average sound absorption coefficient are calculated. Table 8 shows the results of simulation and actual experimental. The rows of “Experiment” and “Simulation” denote the actual experimental results and simulation results, respectively. The row of “Error” denotes the results deviation between the simulation and actual experiments.

It can be seen from Table 8 that the simulation results of MOPSO algorithm are approximately the same as the experimental results. It indicates that MOPSO algorithm has higher accuracy than the GRA method to guarantee the effectiveness of the acoustic package design. According to the comparison, the optimum values for the content of EPDM and deionized water are different. Because the design variables are discrete values in the GRA method and are continuous values in the MOPSO algorithm. Referring to Figure 2, it can be seen that the differences in formulation are responsible for the GRA method having a better sound absorption coefficient and poorer transmission loss than the MOPSO algorithm.

**Figure 3:**
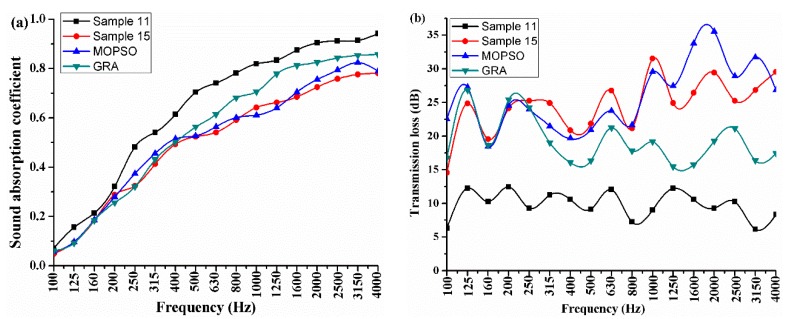
Acoustic performances curve of the PU foam composites (**a**) Sound absorption coefficient curves; and, (**b**) Transmission loss curves.

In addition, Figure 3 shows the acoustic performances curves of the optimized PU foam composites and two initial samples. In Figure 3, “MOPSO” means the PU foam composite prepared according to the formulation that was obtained by the MOPSO algorithm, and “GRA” means the PU foam composite prepared according to the formulation as obtained by the GRA method. Sample 11 has the best average sound absorption coefficient and sample 15 has the best average sound transmission loss. The change trends of the sound absorption coefficient and transmission loss are similar. It can be seen from the results comparison between the MOPSO and sample 15, the average sound absorption coefficient of MOPSO is increased by 2.4% and the average transmission loss is increased by 4.86%. This is possibly due to the optimum values for the content of deionized water, the content and hardness of EPDM are different. However, the difference for the content of deionized water is smaller. Referring to Figure 2, it can be found that the acoustic performances differences of PU foam composites are mainly affected by the content and hardness of EPDM. It agrees with the results that were obtained by GRA method. Moreover, it can be seen that the GRA sample shows better sound absorption coefficient than the MOPSO sample, and the deviation of the sound absorption coefficient of the two samples increase with an increasing frequency. However, the transmission loss of the two samples appears the opposite trend. As shown in Figure 2, 2 g EPDM and 3.5 g deionized water are advantageous for improving the sound absorption ability, and 5.8 g EPDM and 3.4 g deionized water are good for improving the sound insulation ability. In this paper, in order to find the compromise values for these conflicting objectives, the weight coefficient of the two objectives is set as 0.5. Therefore, the optimum formulation of PU foam composites is obtained by the MOPSO algorithm. The optimum values of the four design variables are MDI of 32 g, deionized water of 3.4 g, EPDM of 5.8 g, and the hardness of EPDM is 65 HA.

## 5. Conclusions

In this paper, both GRA method and MOPSO algorithm are used to optimize the synthetic formula of PU foam composites to improve the acoustic performances. The average sound absorption coefficient and average transmission loss are selected as the optimization objectives. The content of MDI and deionized water, the content and hardness of EPDM are selected as design variables. The optimization process of GRA method is based on the orthogonal arrays $L_{9}\left(3^{4}\right)$, and the optimization process of MOPSO algorithm is based on the surrogate model. According to the fitting accuracy comparison, the RS surrogate model is adopted in this paper to express the relation between the optimization objectives and design variables. The results show that the acoustic performances of PU foam composites can be improved by optimizing the formulation of PU foam composites. Meanwhile, the results that were obtained by GRA method show the degree of influence of the four design variables on the optimization objectives. The major influence factor on acoustic performances is the content of EPDM, and the hardness of EPDM has the least influence. The results that were obtained by MOPSO algorithm show the specific effects of the design variables on optimization objectives. However, since the GRA method is usually used to search the optimal solution in discrete spaces, it cannot guarantee the solution is globally optimal solution. Therefore, the optimal results that were obtained by the two optimization methods are different. In this paper, the weight coefficient of the optimization objectives is set as 0.5. By confirmation test, the optimum formulation of PU foam composites is obtained by the MOPSO algorithm. The optimal parameters of the four design variables are MDI of 32 g, deionized water of 3.4 g, EPDM of 5.8 g, and the hardness of EPDM is 65 HA. Certainly, the weight coefficients of the sound absorption ability and sound insulation ability can be set as various values in the range of 0 to 1 to meet different operating conditions requirements.

## Figures and Tables

**Table 1:** Pure polyurethane (PU) foam formulation.

Raw Materials	Content (g)
Polyols (330 N, 3630)	330 N = 60, 3630 = 40
MDI	28–32
Catalyst (A1, A33, TEA)	A1 = 0.05, A33 = 1, TEA = 3
Silicone oil	1.8
Deionized water	2.5–3.5

**Table 2:** Design variables and their levels.

Variables	Parameter Code	Level		
		1	2	3
Content of MDI/g	A	28	30	32
Content of EPDM/g	B	2	4	6
Hardness of EPDM/HA	C	65	70	85
Content of deionized water/g	D	2.5	3	3.5

**Table 3:** Experiment design and experimental results.

Runs	Variables				Average Sound Absorption Coefficient	Average Transmission Loss/dB
	A	B	C	D		
1	28	2	65	2.5	0.614	12.705
2	28	4	70	3	0.577	14.385
3	28	6	85	3.5	0.573	16.792
4	30	2	85	3	0.511	20.887
5	30	4	65	3.5	0.574	16.298
6	30	6	70	2.5	0.580	13.991
7	32	2	70	3.5	0.521	22.272
8	32	4	85	2.5	0.557	18.445
9	32	6	65	3	0.524	19.826
10	28	4	65	3	0.607	10.762
11	28	6	65	2.5	0.637	9.789
12	30	4	85	3	0.543	19.906
13	30	6	70	3	0.519	21.445
14	32	6	85	3	0.528	20.175
15	32	4	70	3.5	0.507	24.570

**Table 4:** Calculation results of normalized sequences, grey relational coefficient and grey relational grade (GRG).

Runs	Normalized Sequences		Grey Relational Coefficient		GRG
	Average Sound Absorption Coefficient	Average Transmission Loss	Average Sound Absorption Coefficient	Average Transmission Loss/dB	
1	1.000	0.000	1.000	0.333	0.667
2	0.641	0.176	0.582	0.378	0.48
3	0.602	0.427	0.557	0.466	0.512
4	0.000	0.855	0.333	0.775	0.554
5	0.612	0.376	0.563	0.445	0.504
6	0.670	0.134	0.602	0.366	0.484
7	0.097	1.000	0.356	1.000	0.678
8	0.447	0.600	0.475	0.556	0.516
9	0.126	0.744	0.364	0.661	0.513

**Table 5:** Calculation results of average GRG.

Variables	Average GRG			Range
	Level 1	Level 2	Level 3	
A	0.553	0.514	0.569	0.055
B	0.633	0.5	0.503	0.13
C	0.561	0.547	0.527	0.034
D	0.556	0.516	0.565	0.049

**Table 6:** Experimental sample for accuracy evaluation of surrogate models.

Runs	Variables				Average Sound Absorption Coefficient	Average Transmission Loss/dB
	A	B	C	D		
1	30	2	65	3	0.567	15.912
2	30	6	65	3	0.551	17.914
3	30	4	70	3	0.533	19.635
4	30	4	65	3	0.561	16.212
5	32	2	85	3	0.481	23.801

**Table 7:** Evaluation coefficients of the surrogate models.

Objectives	Surrogate Models	DC	RAAE	RMAE
**Average sound absorption coefficient**	RS model	0.9507	0.0664	0.1402
	Kriging model	0.6440	0.1521	0.4399
	RBFNN model	0.8073	0.1203	0.3115
**Average transmission loss**	RS model	0.9653	0.0583	0.0968
	Kriging model	0.6793	0.1846	0.3280
	RBFNN model	0.9229	0.0810	0.1695

**Table 8:** Optimization results of simulation and actual experimental.

Methods		Content of MDI/g	Content of EPDM/g	Hardness of EPDM/HA	Content of Deionized Water/g	Average Sound Absorption Coefficient	Average Transmission Loss/dB
GRA	Experiment	32	2	65	3.5	0.552	20.221
	Simulation	32	2	65	3.5	0.532	21.666
	Error	——	——	——	——	−0.02	1.445
MOPSO	Experiment	32	5.8	65	3.4	0.519	25.764
	Simulation	32	5.8	65	3.4	0.512	25.85
	Error	——	——	——	——	−0.007	0.086
